# Epidural Hematoma: The Outcome of Glucocorticoids’ Complementary Use to Surgical Treatment

**DOI:** 10.7759/cureus.22607

**Published:** 2022-02-25

**Authors:** Ekaterini Kourouklari, Demetris Charalambous, Konstantinos Faropoulos, George Fotakopoulos

**Affiliations:** 1 Neurological Surgery, Rheinisch-Westfälische Technische Hochschule Aachen, Aachen, DEU; 2 Neurosurgery, Nicosia General Hospital, Nicosia, CYP; 3 Neurosurgery, General University Hospital of Larisa, Larisa, GRC

**Keywords:** case study, complementary treatment, surgical treatment, dexamethasone, epidural hematoma

## Abstract

Background: The use of glucocorticoids in trauma patients with parenchymal damage is deemed unnecessary and is not advocated. Notwithstanding, acute epidural hematomas (aEH) are extra-parenchymal lesions, so the patients could benefit from the use of glucocorticoids.

Methodology/Results: 97 patients with acute epidural hematoma were separated into two groups, whether they received glucocorticoid treatment or not. Depending on the severity of the deficit and their clinical status, some of the patients were operated on and others not. The patients who received glucocorticoids had better neurological status upon discharge, while their hospitalization was shorter.

Conclusions: The surgical management of the acute epidural hematomas in combination with glucocorticoid treatment had the best outcome in our protocol.

## Introduction

Epidural hematomas are a leading cause of death and disability, especially within the age group of 45 years old and under [1,2]. The application of glucocorticoids in traumatic-induced hemorrhages, mainly through the iv route, was used by some during the previous decades, but it has been discontinued due to its ineffectiveness and complications like Cushing’s syndrome, gastrointestinal ulcers, or higher case mortalities [3-7].

All these research protocols about the use of glucocorticoids in trauma were included in cases with severe parenchymal damage. On the other hand, epidural hematomas are extraparenchymal lesions; thus, the damage they inflict is due to direct pressure on the brain. Additionally, the use of glucocorticoids for other extraparenchymal lesions is well established. So, there is scientific consensus regarding the beneficial role of the latter in meningiomas [8-11], as it helps reduce the volume of the edema due to unknown mechanisms. Furthermore, in chronic subdural hematomas, where there is no apparent damage to the brain parenchyma, glucocorticoids are effective in low doses for a short period [12-14]. One potential mechanism is that glucocorticoids impede the formation of neo-membranes and neo-capillaries [12].

The aim of this research protocol is to elucidate a possible supplementary role of glucocorticoids in the surgical treatment of epidural hematomas, even though the exact mechanism is yet to be understood. Additionally, demographic data, medical history, and trauma characteristics were compared in the population included in the study for the purpose of identifying potential prognostic factors for the outcome, whether the patients were treated surgically or conservatively.

## Materials and methods

**Figure 1:**
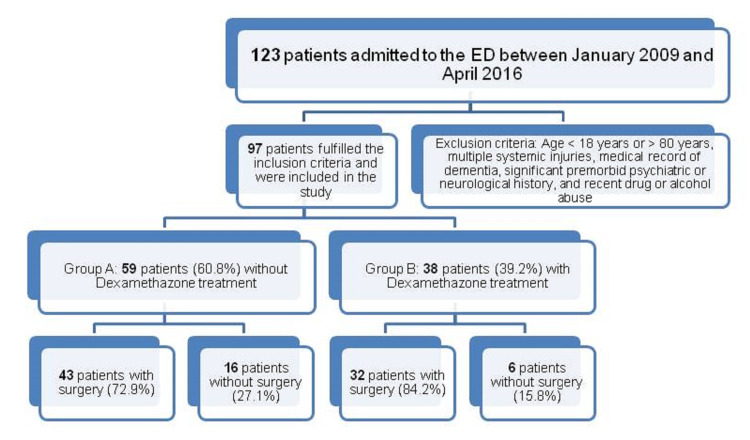
Study selection process. The total number of patients who were hospitalized during the study period and were screened for eligibility.

All patients with an epidural hematoma who presented to the emergency department (ED) between January 2009 and April 2016 (a period of 88 months) were evaluated retrospectively. Exclusion criteria included age <18 years or >85 years, multiple systemic injuries, a medical record of dementia, a significant premorbid psychiatric or neurological history, and recent drug or alcohol abuse (Figure 1). 

**Figure 2:**
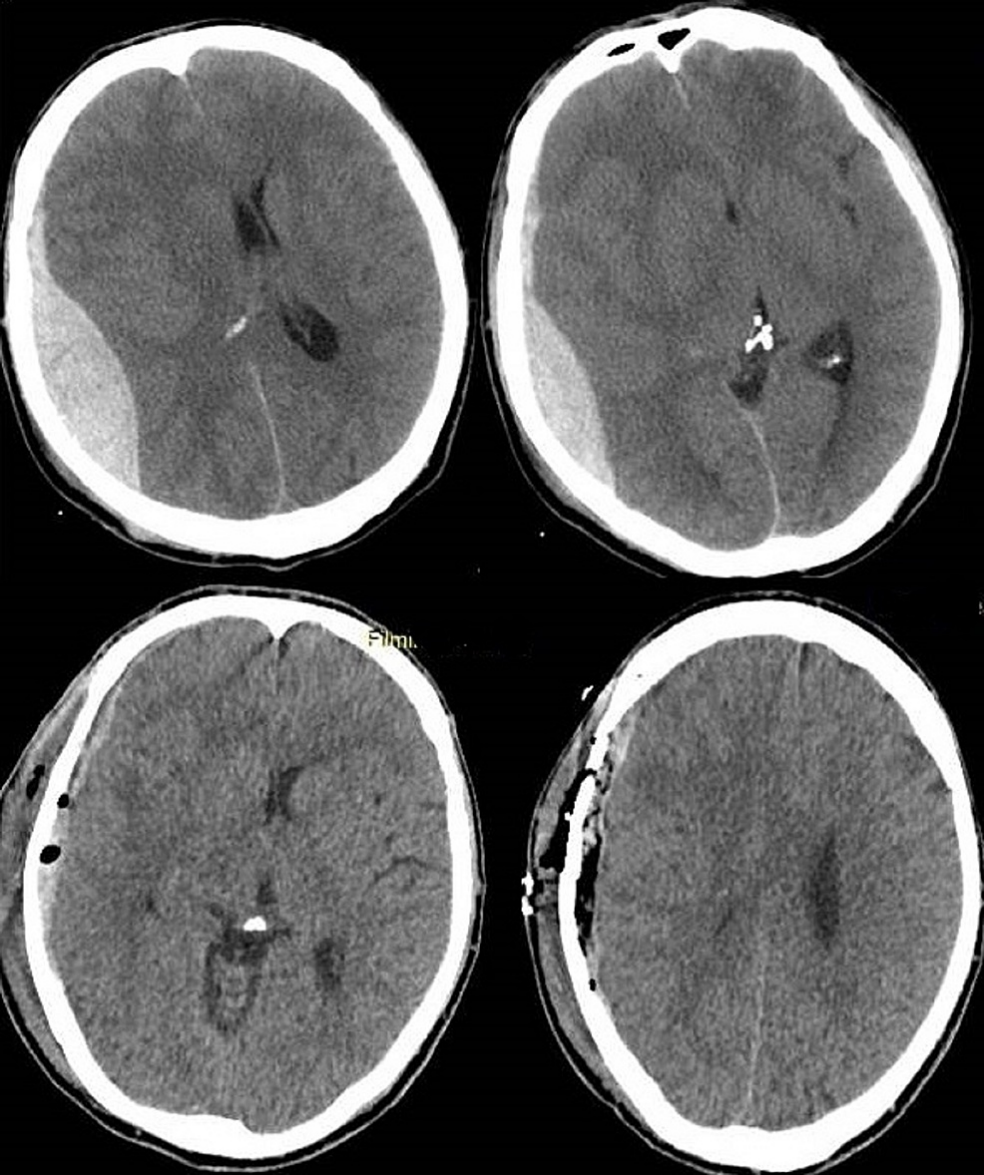
CT scan Acute epidural hematoma before and after evacuation

Patients who were included in the study were classified into two groups: those who started intravenous dexamethasone therapy during the first 24 hours of admission and those without dexamethasone treatment (patients with uncontrollable diabetes, ulcers, or infection). Both groups contained patients with variant neurological deficits, while the inclusion criteria for each group was not the severity of the deficit but the eligibility for a glucocorticoid treatment. The dose was 4 mg, three times per day for one week, and then gradually reduced every three days and discontinued for seven days. For the patients who needed surgical evacuation of the epidural hematoma, the operation was performed during the first 48 hours of hospitalization, using a standard craniotomy, tailored in accordance with the location of the hematoma (Figure 2). That treatment was offered to patients with neurological deficits, rapid deterioration, or extensive hematomas on the CT scan. Surgical treatment was not offered to patients with small hematomas below 0.8 cm in the CT scan, mild symptomatology, significant comorbidity who could not tolerate a craniotomy, or patients who selected to be treated non-operatively, despite our strong recommendation for surgical treatment.

Patients continued their follow-up at one, six, and twelve months after the incident. All the patients, upon their arrival, were informed about the potential complications of the treatment with glucocorticoids and signed consent was obtained, while they were treated in accordance with the tenets of the Declaration of Helsinki (as revised in Edinburgh 2000).

Outcomes

In our study, the results obtained were based on two factors: the one being the recurrence of a neurological deficit after the incident, and the other the total number of days of hospitalization. A good outcome is defined as nine or fewer days of hospitalization, which derives from a receiver operator characteristic (ROC) analysis.

Clinical data

The patients were examined for motor deficits upon their arrival at the hospital, during their hospitalization, before exiting from the hospital, and during their follow-up in the outpatient clinic. They had different forms of motor neurological deficits initially, ranging from mild monoparesis (4/5 muscle strength) to hemiplegia (0/5 muscle strength). Central nervous system (CNS) pathology was examined based on medical history and computed tomography (CT) scan, alcohol consumption was assessed by the Alcohol Use Disorders Identification Test-Consumption (AUDIT-C) [15] and drug consumption was assessed by drug abuse testing using a 24-hour urine or serum sample [2].

In all cases, multiple parameters like Glasgow Coma Scale (GCS) at the time of admission, trauma mechanism (car/motorcycle accident, fall, and assault) (Table 1), sex, age, and medical history (hypertension, coronary heart disease, chronic smokers, diabetes, anticoagulation/antiplatelet medication, brain surgery or traumatic brain injury in the past, and seizures) and previous hospitalization were documented (Table 1). These parameters were taken under statistical analysis in order to determine if they could be used as predictive markers of the outcome. Then, patients were divided into two groups accordingly; group A: those who had not received dexamethasone treatment; and group B: those who had started dexamethasone therapy on time. We have chosen to include in group A patients with contraindications to glucocorticoids, like infection, poorly controlled diabetes, recent gastrointestinal bleeding or ulcer, and in group B patients who could tolerate glucocorticoid treatment.

**Table 1:** Baseline characteristics of patients suffering a moderate traumatic brain injury Data are presented as mean ± SD, otherwise is indicated. Abbreviations: standard deviation (SD), GCS: Glasgow Coma Scale, RTS: Revised Trauma Score, P-value for the difference between groups was assessed for Nominal data using the Fisher’s exact test and for continuous data with the Mann-Whitney U-test as appropriate.

Parameters	All patients n=97	Group A: n=59(60.8%)	Group B: n=38(39.2%)	P-value
Age, years	40.8±13.7	41.6±12.8	39.4±14.9	0.282
Sex (male), n(%)	48, (49.4)	27, (45.7)	21, (55.2)	0.409
GCS of admission, mean±SD	11.5±1.8	11.5±1.8	11.5±1.7	0.916
Surgery/no surgery(surgery), n(%)	75(77.3)	43(44.3)	32(32.9)	0.223
Hypertension, n(%)	20(20.6)	12(12.3)	8(8.2)	0.564
Coronary heart disease, n(%)	7(7.2)	5(5.1)	2(2.1)	0.434
Chronic smokers, n(%)	28(28.8)	19(19.5)	9(9.2)	0.252
Diabetes, n(%)	9(9.2)	5 (5.1)	4(4.1)	0.498
History of brain surgery or head injury, n(%)	4(4.1)	2(2.1)	2(2.1)	0.512
Seizures, n(%)	4(4.1)	2(2.1)	2(2.1)	0.512
Anticoagulation/antiplatelet treatment, n(%)	6(6.1)	4(4.1)	2(2.1)	0.562
RTS, mean±SD	7.243±0.3	7.249±0.7	7.342±0.6	0.128
Hospital stays in days, mean±SD	12.2±04.4	13.9±4.6	9.7±2.7	0.000

Statistical analysis

Data are expressed as mean ± standard deviation (SD). Data were assessed for normality using the Shapiro-Wilkes test. Nominal data were analyzed using the Fisher’s exact test. Continuous data were analyzed using the Student’s t-test or the Mann-Whitney U-test as appropriate. Variables significantly associated with univariate analysis were then entered into a multivariable analysis model. A p-value < 0.05 was considered statistically significant. Statistical analyses were performed with the use of Statistical Product and Service Solutions (SPSS) software, version 15 (SPSS Inc., Chicago, IL, USA).

## Results

There were 97 out of 123 patients with acute epidural hematoma (aEDH) after a traumatic brain injury (TBI) (males: 48, 49.4%) who fulfilled the study inclusion criteria (Table 1). There were 59 patients (60.8%) without dexamethasone treatment (group A) and 38 (39.2%) who received dexamethasone (group B).

The patients preoperative status varied from GCS 15 to comatose patients. Patients in group B presented significant differences compared to group A in terms of hospital stay and recovery from the neurological deficit (Table 2). Among patients who underwent craniotomy (n=75), 41/43 patients in group A had no neurological deficit, and 32/32 patients in group B had no neurological deficit. Among patients who did not undergo craniotomy, 12/16 in group A had no neurological deficit compared to 6/6 in group B (p = 0.046). Thus, all patients in the glucocorticoid group had no neurological deficit, a significantly improved outcome compared to group A for both operative and non-operative comparisons (Table 2).

**Table 2:** Patients' outcomes Data are presented as mean ± SD, otherwise is indicated

Outcomes	Surgery, n=75	No surgery, n=22	P-value
Outcome 1: no neurological deficit			
Group A, n (%)	41 from 43 (69.4)	12 from 16 (20.3)	0.046
Group B, n (%)	32 from 32 (84.2)	6 from 6 (15.8)	
Outcome 2: hospital stay in days			
Group A, n (%)	10/16 (16.9)	4/43 (6.7)	0.001
Group B, n (%)	1/6	21/32	

The second outcome (nine or fewer days of hospitalization) was 14 patients from group A (10 with surgery and 4 without surgery) and 22 from group B (1 with surgery and 21 without surgery; P-value = 0.001) (Table 2). Thus, dexamethasone has helped reduce the number of hospital days greatly (57.8% in group B compared to 27.1% in group A). Moreover, there was a significant difference between those patients with surgery and those with no surgery in terms of GCS of admission, chronic smokers, and days of hospitalization (p<0.05; Table 3).

**Table 3:** Comparison between patients with surgery/no surgery Data are presented as mean ± SD, otherwise is indicated. Abbreviations: standard deviation (SD), GCS: Glasgow Coma Scale, P-value for the difference between groups was assessed for nominal data using the Fisher’s exact test and for Continuous data with the Mann-Whitney U-test as appropriate.

Parameters/N=97	Surgery, n=75(%)	No Surgery, n=22(%)	P-value
Age, years	40.3±13.5	42.2±14.3	0.730
Sex (male), n (%)	33(34.0)	15(15.4)	0.055
GCS of admission, mean ± SD	11.1±1.7	13.0± 0.9	0.000
Hypertension, n (%)	15(15.4)	5(5.1)	0.495
Coronary heart disease, n (%)	4(4.1)	3(3.1)	0.190
Chronic smokers, n (%)	17(17.5)	11(11.3)	0.015
Diabetes, n (%)	7(7.2)	2(2.1)	0.668
History of Brain surgery or head injury, n(%)	3(3.1)	1(1.0)	0.649
Anticoagulation/antiplatelet treatment, n(%)	3 (3.1)	3(3.1)	0.128
Hospital stays in days, mean ± SD	12.9±4.7	9.9±1.6	0.013
Groups			
Group B	32(32.9)	6(6.1)	0.223
Group A	43(44.3)	16(16.4)	

Outcomes

Clinical outcomes are shown in Table 2 and only hospital stay was significantly associated with groups (P<0.05). Overall, the patients who received dexamethasone treatment spent fewer days in the hospital, and the number of patients who were hospitalized for nine days or less was relatively higher in group B in comparison to group A (P<0.05; Table 2).

**Figure 3:**
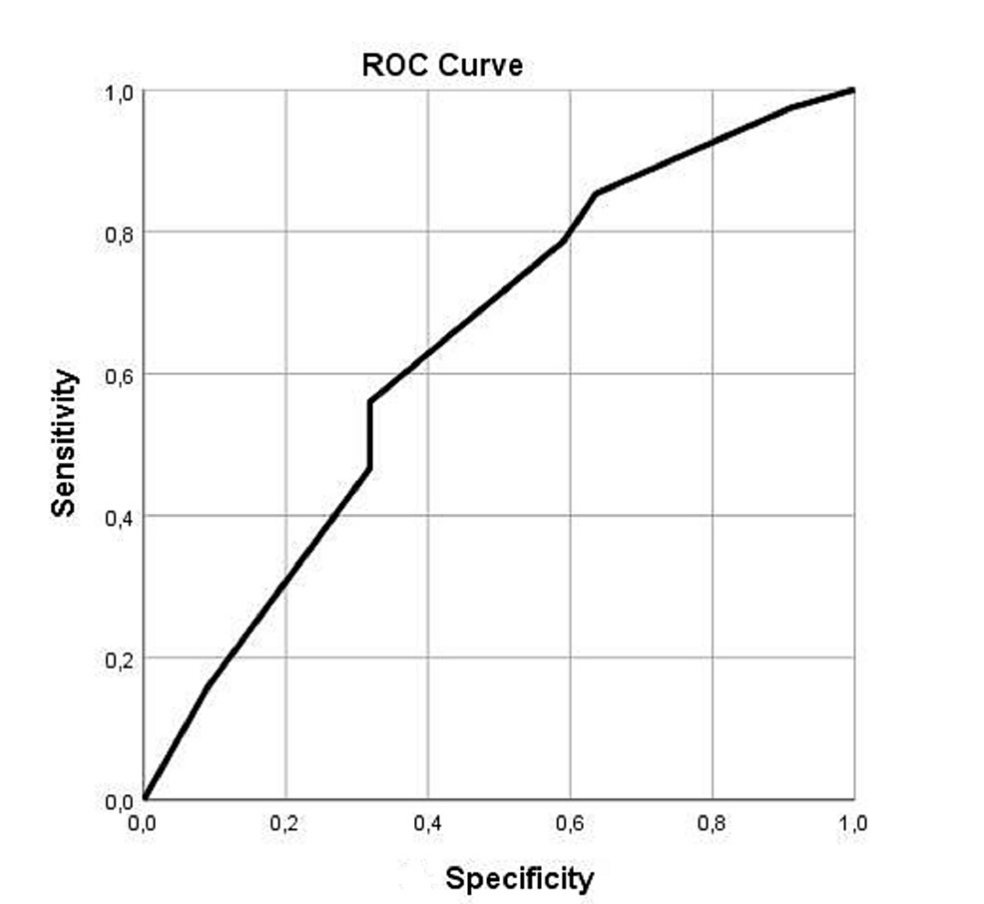
Receiver operator characteristic curve ROC curve for GCS of admission. ROC: receiver operator characteristic, GCS: Glasgow Coma Scale.

**Figure 4:**
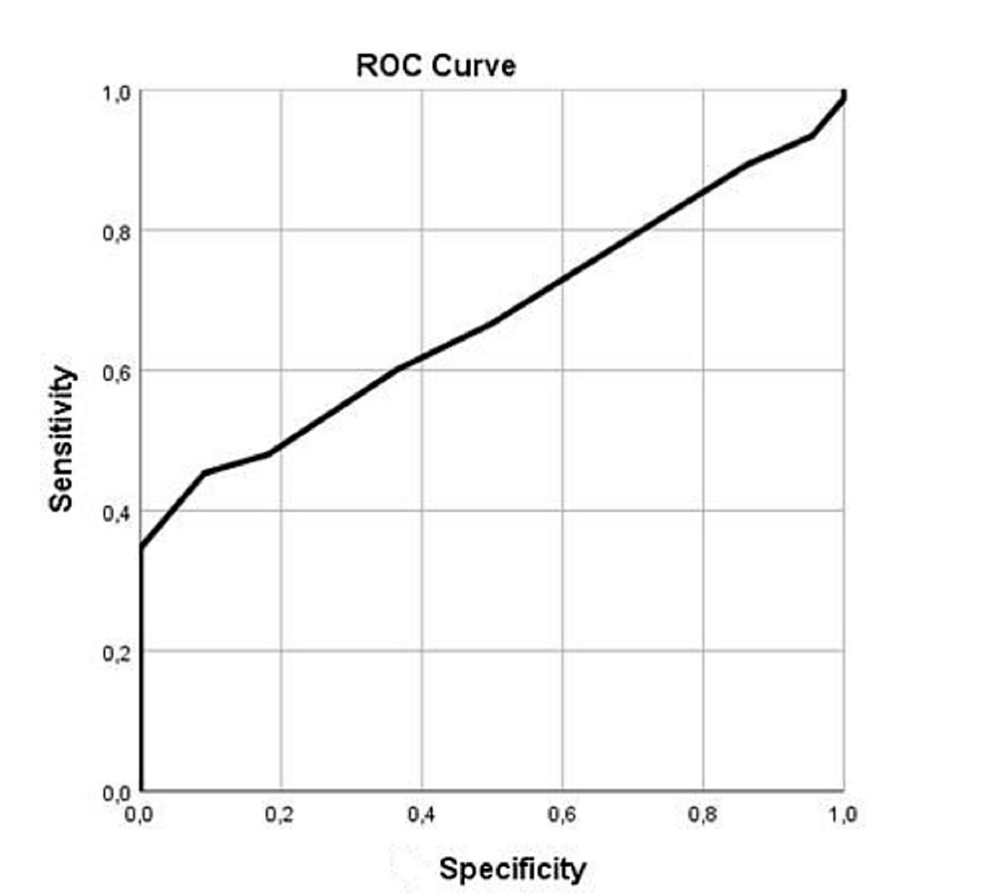
Receiver operator characteristic curve ROC curve for hospital stay. ROC: receiver operator characteristic.

ROC analysis showed that hospital stay and GCS of admission presented significant values for patients with surgery and no surgery [sensitivity of 89% and specificity of 86% with the best performance of 8.5 days (95% confidence interval (CI) 0.700-0883), p=0.014] and [sensitivity of 70% and specificity of 63% with the best performance a value of GCS=12 (95% confidence interval (CI) 0.500-0776), p=0.050], respectively (Table 4; Figures 3-4).

**Table 4:** Statistical findings for receiver operator characteristics Data are presented as n(%), otherwise is indicated. Abbreviations: Std: standard, CI: confidence interval, GCS: Glasgow Coma Scale.

Parameters	Area	Std error	CI (95%) lower-upper	P-value
Groups	0.557	0.059	0.441–0.673	0.348
Chronic smokers	0.637	0.071	0,498–0.775	0.052
GCS of admission	0.638	0.070	0.500–0776	0.050
Hospital stay	0.674	0.055	0.567–0.781	0.014

## Discussion

The use of corticosteroids in the case of epidural hematoma is a controversial issue. If we would like to highlight the main findings of the present retrospective study, they would be: (a) patients in group B spent fewer days in the hospital; (b) patients in group B who had surgery had a better outcome than those who did not within the same group; (c) the GCS of admission for the patients who underwent surgery played a crucial role in the outcome; and (d) hospital stay in patients with surgical treatment and glucocorticoid usage was less.

In more detail, all patients treated with dexamethasone had a shorter hospitalization period. Additionally, the patients who were treated with dexamethasone and underwent an operation had a better outcome than those who did not within the same group (57.8% vs 27.1%). These outcomes serve as an indication of the advantageous results of dexamethasone therapy and operative treatment. Moreover, for patients who underwent surgery, the GCS of admission plays a crucial role in the outcome with 70% sensitivity and 63% specificity with the best performance of the ROC Curve at a value of GCS = 12 (95% confidence interval (CI) 0.500-0776)], p=0.050]. This means that patients with a GCS=12 who underwent surgery and received dexamethasone have shown the best results. Finally, the duration of hospitalization in patients with surgical treatment was shorter [sensitivity of 89% and specificity of 86% with the best performance at 8.5 days (95% confidence interval (CI) 0.700-0883)], p=0.014].

Our findings seem to be in conflict with previous research protocols, where the use of steroids in traumatic brain injuries was deemed not only ineffective [16] but was correlated with significant complications [3-7].

On the other hand, epidural hematomas are located outside the dura mater; therefore, the damage caused is due to pressure, as they do not directly encounter brain parenchyma. Other extraparenchymal lesions are meningiomas and chronic subdural hematomas. In both cases, the use of glucocorticoids has been clinically proven to be of an advantage, as in meningiomas they help reduce the perifocal vasogenic edema that surrounds the primary lesion [17], while in the latter they inhibit neo-angiogenesis [12]. However, glucocorticoids may have an additional effect mechanism. In meningiomas, a correlation between edema and prostaglandins is also possible [18]. It is known that glucocorticoids interfere with the inflammation cascade. The binding of the hormone on its cytoplasmic receptor induces, through a signal pathway, the activation of Lipocortin-1, which has been observed to down-regulate phospholipase A2. Phospholipase A2 is responsible for the cleavage of arachidonic acid (AA) from cellular membrane-forming phospholipids [15,19]. Furthermore, glucocorticoids affect the production of prostaglandins by blocking the enzymes that are directly involved in the metabolization of AA, such as cyclooxygenase/PGE isomerase (COX-1 and COX-2) [20,21].

An inflammatory response is thought to be a contributing factor in the formation of chronic subdural hematomas. So, glucocorticoids inhibit inflammatory mediators such as lymphokines and prostaglandins and stimulate inflammatory inhibitors like lipocortin and, through that, neovascularization [12].

Balancing the proinflammatory factors or inhibiting the inflammatory response through glucocorticoids could accelerate the recovery of the neurological deficit and, as a result, reduce the hospitalization period. Alternatively, cortisone is also known to play a serviceable role in reducing the levels of stress hormones in the organism. As in epidural hematoma, the damage is to the parenchyma, so the reduction of stress hormones could accelerate the recovery of the brain and, consequently, the motor deficit.

It should be underlined here that there are some points that should be taken into consideration in the interpretation of our results. First, patients over 85 or less than 18 years old were excluded from the study, and therefore, our results may not be valid in these age groups. Older patients were excluded because they may often present with cognitive problems and disabilities due to pathological causes that could distort our findings. On the other hand, patients less than 18 years old present different recovery rates and durations compared to older patients, which could reduce the homogeneity in the study population. Another limitation of this study is the fact that it is a single-center study with a limited but statistically important number of patients included. Additionally, due to the limited number of patients, the two groups could not be balanced for gender, coagulation, or forms of comorbidity. Also, the reason why these comorbidities did not interfere with the less favorable outcome of group A may have been that they prolonged hospitalization but did not prevent neurological recovery. It is possible that a larger cohort or a multicenter study could provide solid evidence supporting the complementary role of glucocorticoids in the management of epidural hematomas.

## Conclusions

Patients who underwent surgical management of the epidural hematoma and received additional dexamethasone treatment had a rapid neurological recovery and shorter hospitalization. Thus, the use of dexamethasone as a complementary therapy to the surgical treatment of an epidural hematoma could be beneficial if there was no contradiction in its use.

## References

[1] Clarici GC (2017). [Surgical techniques for severe brain injury: with special emphasis on polytrauma]. Unfallchirurg.

[2] Haselsberger K, Pucher R, Auer LM (1988). Prognosis after acute subdural or epidural haemorrhage. Acta Neurochir (Wien).

[3] Edwards P, Arango M, Balica L (2005). Final results of MRC CRASH, a randomised placebo-controlled trial of intravenous corticosteroid in adults with head injury—outcomes at 6 months. Lancet.

[4] Martino EA, Baiardo Redaelli M, Sardo S (2018). Steroids and survival in critically Ill adult patients: a meta-analysis of 135 randomized trials. J Cardiothorac Vasc Anesth.

[5] Oray M, Abu Samra K, Ebrahimiadib N, Meese H, Foster CS (2016). Long-term side effects of glucocorticoids. Expert Opin Drug Saf.

[6] Roberts I, Yates D, Sandercock P (2000). Effect of intravenous corticosteroids on death within 14 days in 10008 adults with clinically significant head injury (MRC CRASH trial): randomised placebo-controlled trial. Lancet.

[7] Schacke H (2002). Mechanisms involved in the side effects of glucocorticoids. Pharmacol Ther.

[8] Dietrich J, Rao K, Pastorino S, Kesari S (2011). Corticosteroids in brain cancer patients: benefits and pitfalls. Expert Rev Clin Pharmacol.

[9] Heiss JD, Papavassiliou E, Merrill MJ (1996). Mechanism of dexamethasone suppression of brain tumor-associated vascular permeability in rats. Involvement of the glucocorticoid receptor and vascular permeability factor. J Clin Invest.

[10] Kaal EC, Vecht CJ (2004). The management of brain edema in brain tumors. Curr Opin Oncol.

[11] Murayi R, Chittiboina P (2016). Glucocorticoids in the management of peritumoral brain edema: a review of molecular mechanisms. Childs Nerv Syst.

[12] Delgado-López PD, Martín-Velasco V, Castilla-Díez JM, Rodríguez-Salazar A, Galacho-Harriero AM, Fernández-Arconada O (2009). Dexamethasone treatment in chronic subdural haematoma. Neurocirugía.

[13] Henaux PL, Le Reste PJ, Laviolle B, Morandi X (2017). Steroids in chronic subdural hematomas (SUCRE trial): study protocol for a randomized controlled trial. Trials.

[14] Kolias AG, Chari A, Santarius T, Hutchinson PJ (2014). Chronic subdural haematoma: modern management and emerging therapies. Nat Rev Neurol.

[15] Goppelt-Struebe M, Wolter D, Resch K (1989). Glucocorticoids inhibit prostaglandin synthesis not only at the level of phospholipase A2 but also at the level of cyclo-oxygenase/PGE isomerase. Br J Pharmacol.

[16] Müller W, Kretzschmar K, Schicketanz KH (1984). CT-analyses of cerebral tumors under steroid therapy. Neuroradiology.

[17] Lambertz N, Hindy NE, Adler C (2013). Expression of aquaporin 5 and the AQP5 polymorphism A(-1364)C in association with peritumoral brain edema in meningioma patients. J Neurooncol.

[18] Constantini S, Tamir J, Gomori MJ, Shohami E (1993). Tumor prostaglandin levels correlate with edema around supratentorial meningiomas. Neurosurgery.

[19] Buckingham JC (2006). Glucocorticoids: exemplars of multi-tasking. Br J Pharmacol.

[20] Nakano T, Ohara O, Teraoka H, Arita H (1990). Glucocorticoids suppress group II phospholipase A2 production by blocking mRNA synthesis and post-transcriptional expression. J Biol Chem.

[21] Revollo JR, Cidlowski JA (2009). Mechanisms generating diversity in glucocorticoid receptor signaling. Ann N Y Acad Sci.

